# Efficacy of intragastric botulinum toxin A injection on patients with side effects caused by intragastric balloon placement intolerance: A case series study

**DOI:** 10.1097/MD.0000000000041411

**Published:** 2025-03-14

**Authors:** Farzad Faramarzi Garousi, Mohsen Sedighiyan, Maryam Ghodsi

**Affiliations:** a Department of Gastroenterology, Imam Hossein Hospital, Shahid Beheshti University of Medical Sciences, Tehran, Iran; b Department of Clinical Nutrition, Bahrami Hospital, Tehran University of Medical Science, Tehran, Iran; c Department of Pediatrics, School of Medicine, Bahrami Hospital, Tehran University of Medical Science, Tehran, Iran.

**Keywords:** botulinum toxin A, gastrointestinal intolerance, intragastric balloon, obesity management

## Abstract

**Rationale:**

Obesity is a global health concern, with intragastric balloon (IGB) placement serving as a nonsurgical intervention for weight management. However, intolerance due to severe gastrointestinal side effects often leads to premature removal, limiting its effectiveness. Botulinum toxin A (BTX-A) injection has been proposed as a potential strategy to improve IGB tolerance by modulating gastric motility.

**Patient concerns:**

This study reports a case series of patients who developed severe intolerance to IGB placement, including persistent nausea, vomiting, fluid intolerance, dehydration, and reduced urine output, necessitating medical intervention.

**Diagnoses:**

Patients were diagnosed with IGB intolerance due to obstructive gastric effects, characterized by impaired gastric emptying and intolerance to oral fluids, leading to dehydration and electrolyte imbalances.

**Interventions:**

A total of 14 patients with IGB intolerance were treated with 500 U of BTX-A injected around the pyloric canal. Prokinetic drugs were also administered to support gastric motility. Patients were monitored for 6 months following the intervention.

**Outcomes:**

Among the 14 patients, 11 (78.57%) showed a significant improvement in fluid tolerance within 12 hours of BTX-A injection and were able to retain the IGB without additional complications. Three patients (21.43%) did not improve and required early IGB removal. No major adverse effects related to BTX-A injection were observed.

**Lessons:**

BTX-A injection appears to be a promising adjunctive therapy to improve IGB tolerance in patients experiencing severe intolerance. These findings suggest a potential role for BTX-A in reducing the need for premature balloon removal, thereby enhancing weight loss outcomes. However, further randomized controlled trials with larger sample sizes are needed to confirm its efficacy, optimize dosage, and establish standardized treatment protocols.

## 1. Introduction

Obesity ranks among the most critical global health challenges of the 21st century, reaching unprecedented prevalence and posing a significant threat to worldwide public health.^[1,2]^ Beyond its detrimental impact on quality of life, obesity is a well-established risk factor for a multitude of chronic diseases, particularly those affecting the cardiovascular, musculoskeletal, and endocrine systems. The World Health Organization reports that worldwide obesity has nearly tripled since 1975, with more than 650 million adults classified as obese in 2016. This alarming trend is associated with an increased risk of developing chronic diseases such as type 2 diabetes mellitus, cardiovascular diseases and certain cancers.^[3–5]^

The health burden associated with obesity translates into significant economic costs for healthcare systems globally. Effective strategies for obesity prevention primarily focus on dietary behavior modification and promoting physical activity.^[6]^ Treatment options for established obesity encompass medical nutrition therapy, pharmacological interventions, psychological counseling, and a range of surgical and nonsurgical endoscopic procedures.^[7,8]^ Achieving sustainable weight loss in obesity necessitates a focus on establishing a negative energy balance, often requiring a reduction in caloric intake for the majority of patients. However, dietary interventions for obesity management are plagued by concerningly high rates of long-term nonadherence, hovering around 80% to 90%.^[9]^

Intragastric balloon (IGB) placement has emerged as a nonsurgical weight loss intervention for patients with a body mass index (BMI) between more than 27 kg/m² who have failed conservative measures.^[10]^ IGB placement has emerged as an effective, nonsurgical intervention for weight loss in patients with obesity. This procedure involves the endoscopic placement of a saline-filled silicone balloon within the stomach, which induces satiety and reduces food intake by occupying gastric volume.^[11]^ Clinical trials and meta-analyses have demonstrated the efficacy of IGB in achieving significant weight loss and improving metabolic parameters.^[12]^ However, despite its benefits, IGB placement is frequently accompanied by adverse gastrointestinal symptoms, including nausea, vomiting, abdominal pain, dehydration, and gastroesophageal reflux.^[13]^ These side effects can lead to IGB intolerance, necessitating early removal of the balloon in approximately 3% to 6% of patients. The high incidence of IGB intolerance poses a considerable challenge, potentially undermining the therapeutic benefits of the procedure.^[14]^ Recent advancements in gastroenterological interventions have identified intragastric botulinum toxin A (IGBTA) injection as a promising adjunctive treatment to enhance patient tolerance to IGB. Botulinum toxin A, derived from *Clostridium botulinum*, is a potent neurotoxin known for its ability to inhibit acetylcholine release at neuromuscular junctions, leading to muscle relaxation.^[15]^ This mechanism has been effectively utilized in the treatment of various conditions, including dystonia, spasticity, and chronic migraine. In the context of gastrointestinal applications, IGBTA has been shown to modulate gastric motility and reduce visceral hypersensitivity, which may alleviate the gastrointestinal symptoms associated with IGB intolerance.^[16]^

This case series study aims to investigate the efficacy of intragastric BTX-A injection in patients experiencing severe side effects due to IGB placement. By providing a detailed account of clinical outcomes, symptom resolution, and patient tolerance, we seek to contribute to the growing body of evidence supporting the use of BTX-A as a therapeutic intervention for IGB intolerance.

## 2. Materials and methods

**Figure 1.**
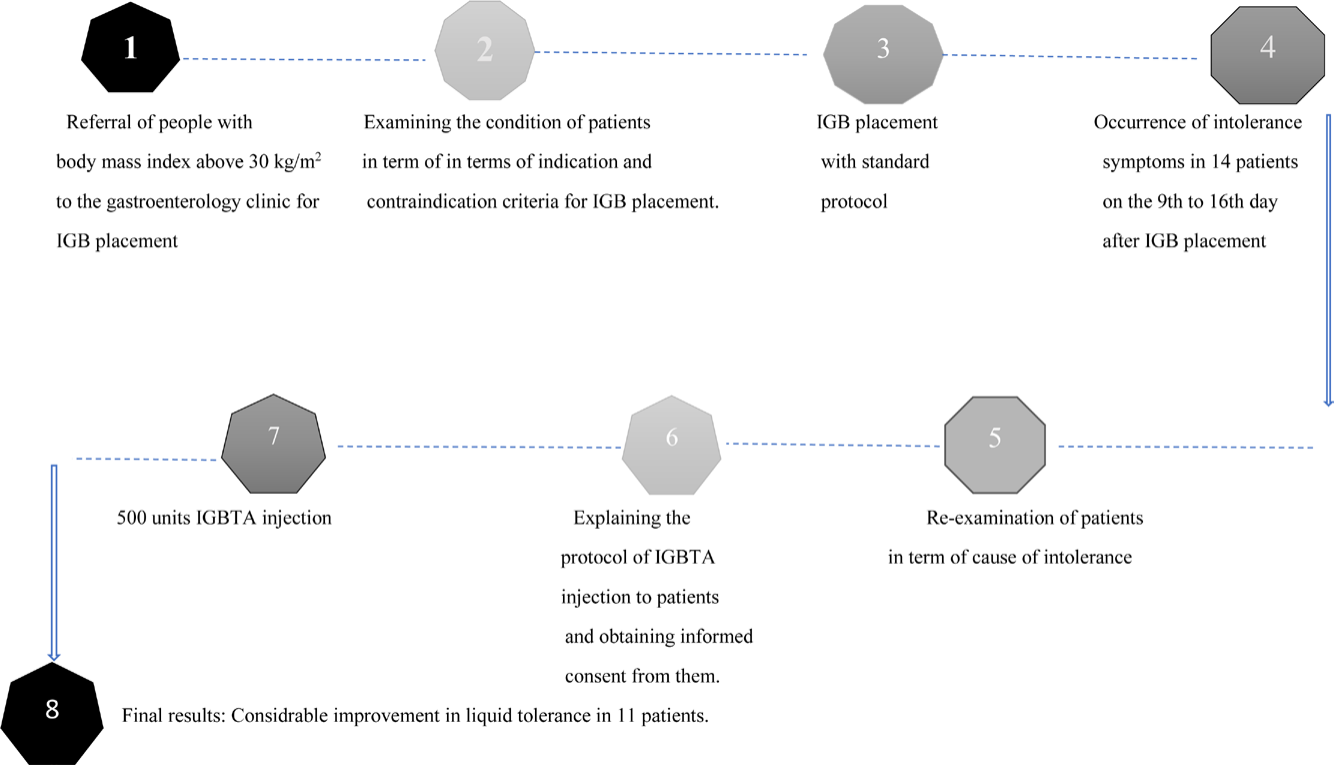
Timeline of events.

Timeline of events was summarized in Figure 1. Our research is a retrospective case series study, which was conducted in Gastroenterology Clinic in Tehran University of Medical Sciences, Iran, between the 2022 and 2024 April. All patients were provided with written and verbal information about the study and informed consent was obtained from all subjects. The process of IGB in this clinic is performed by an expert gastroenterologist. The patients were informed of the treatment methods before application. In this specialized clinic, the protocol of IGB was performed based on standard protocol for patients with BMI more than 27 kg/m^2^, without any malignancy, autoimmune diseases, certain allergies and previous major abdominal surgery. Absolute contraindications for performing IGB in this center included: history of gastrointestinal tumor and surgery, coagulation disorders, active bleeding lesion in the upper GI tract, pregnancy or desire to become pregnant, alcoholism or drug addiction, sever liver disease and contraindications to endoscopy. Also, there were some relative contraindications including previous abdominal surgery, large hiatal hernia, inflammatory bowel disease, chronic nonsteroidal anti-inflammatory drug and uncontrolled psychiatric disorder.

According to the established protocol, patients received sedation anesthesia using “Propofol,” a short-acting intravenous agent, administered at a dosage of 1 mg/kg. Prior to proceeding with the procedure, all patients underwent a diagnostic esophago-gastroduodenoscopy (EGD) to evaluate for any potential contraindications. This study employed balloons manufactured by Medsil (CSC MEDSIL, Moskovskaya oblast, Russia) for a period of 6 months for IGB. Upon placement within the stomach, the IGB was inflated with a saline solution containing methylene blue in a concentration ranging from 400 to 600 mL. This inflation marked the completion of the procedure. While the endoscopically measured stomach volume was considered, the inflation solution volume remained within the established range of 400 to 600 mL. To address nausea and intolerance potentially arising from balloon placement, antiemetic therapy was administered. In the first 3 days, ondansetron (8 mg per day) was injected intravenously to the patients, which was injected to the patients with saline solution. Also, patients received 1 Pantoprazole 40 mg powder for solution daily for the first 3 days. Moreover, patients received intravenous paracetamol (Apotel 1 gr). For patients who still had symptoms of nausea after 3 days, ondansetron tablets were prescribed for 1 week. Patients who had balloon intolerance were divided into 2 groups. The first group was the patients whose intolerance caused by the pressure effect of the balloon, whose main symptom is pain and for them the balloons were removed. The second group, which are actually the patients evaluated in this study, intolerance caused by balloon obstructive effect, the main symptom was nausea, vomiting and liquid intolerance.

We used a new method, among patients who presented with obstructive signs and symptoms of balloon intolerance included pressure drop, dehydration, dizziness, decrease in urine volume and electrolyte disorders. The previous approach for these patients, which was used in most clinics and hospitals in the world, includes that this problem is caused by the blockage of the stomach outlet and the balloon should be removed urgently. However, due to the similarity of symptoms and pathophysiology of this problem and patients with refractory gastroparesis, we considered this hypothesis that new treatment for resistant gastroparesis may be effective in treating these patients. Therefore, for the last 14 patients who came with this problem, we used 500 units Massport, which were diluted with a total of 9 cc distilled water and it was injected around the pyloric canal. Massport, in the present study, every 3.5 units of it was equivalent to 1 unit of IGBTA.

Patients were under regular control for 6 months. After performing the recommended procedures, the necessary recommendations were given to the patients and the patients were regularly monitored and the patients were asked to inform the treatment team of any digestive symptoms, change in urine color, lethargy or any other symptoms.

## 3. Results

The baseline characteristics of the patients are summarized in Table 1. This case series included 14 patients aged 18 to 54 years, with BMI values ranging from 30.6 to 61.4. Among the participants, 4 patients had a history of type 2 diabetes, 3 patients had hypertension, and 10 had nonalcoholic fatty liver disease. All patients reported severe intolerance symptoms following IGB placement, including fluid intolerance, dehydration, and decreased urine volume, which were attributed to gastric outlet obstruction caused by the balloon.

**Table 1:** Primary characteristics of the patients.

Patients code	1	2	3	4	5	6	7	8	9	10	11	12	13	14
Sex	Female	Female	Male	Female	Female	Male	Male	Female	Female	Female	Male	Female	Female	Female
Age (yr)	36	32	44	23	35	34	18	54	41	33	34	37	30	40
Weight (kg)	118	93.8	98.7	76.3	133.3	147.2	148.6	102.5	119.8	93.5	112.8	104.9	79.7	81.3
BMI (kg/m^2^)	41.8	31.3	37.9	31.6	43	44.7	61.4	35.4	44.5	37.7	38.3	35.9	39.7	30.6
Diabetes	−	−	−	−	+	+	−	−	+	+	−	−	−	−
High blood pressure	−	−	+	−	−	−	+	+	−	−	−	−	−	−
NAFLD	+	+	−	−	+	+	+	+	+	+	+	+	−	−
Muscular pain	−	−	+	−	−	−	−	+	−	−	−	−	−	−
Botox injection day after the IGB	The 9th day	The 11th day	10th day	The 13th day	The 9th day	The 11th day	16th day	The 12th day	The 14th day	The 11th day	The 13th day	10th day	The 12th day	The 15th day
Liquid intolerance	+	+	+	+	+	+	+	+	+	+	+	+	+	+
Xerostomia	+	+	+	+	+	+	+	+	+	+	+	+	+	+
Decreased urine output	−	+	+	+	+	+	+	+	+	+	+	−	+	+

IGB = intragastric balloon, NAFLD = nonalcoholic fatty liver disease.

Following the injection of 500 units of intragastric botulinum toxin A (BTX-A) around the pyloric canal, 11 patients (78.57%) experienced significant improvement in fluid tolerance within 12 hours. These patients reported no notable side effects following the intervention and were able to retain the IGB for the full 6-month treatment duration. The rapid symptom resolution in this group highlights the potential efficacy of BTX-A in alleviating the gastrointestinal side effects associated with IGB intolerance.

However, 3 patients (21.43%) did not respond to the BTX-A injection. These individuals continued to experience severe symptoms, including persistent vomiting and dehydration, necessitating the early removal of the IGB. This subgroup underscores the variability in response to BTX-A therapy and the need for alternative management strategies in nonresponders.

No adverse effects related to the BTX-A injection were observed in any of the patients during the follow-up period. The intervention was well-tolerated, with no reports of complications such as local infection, allergic reaction, or systemic botulism.

Overall, this case series suggests that BTX-A co-therapy is a promising intervention for managing IGB intolerance, with most patients achieving significant symptom relief and improved treatment outcomes. Further studies with larger sample sizes and control groups are needed to confirm these findings and determine the factors influencing treatment efficacy.

## 4. Discussion

This case series investigated the efficacy of IGBTA injection as a novel therapeutic approach to manage IGB intolerance in patients experiencing severe gastrointestinal side effects. Our findings suggest that IGBTA co-therapy may offer a promising strategy to improve tolerance and potentially enhance the long-term success of IGB therapy for weight management.

Kanlioz et al in a clinical trial evaluated the efficacy of intragastric balloon placement and botulinum toxin injection in bariatric endoscopy. They examined 114 obese people in 3 groups: IGB, IGBTA and the third group including simultaneous treatment with IGB and 100 units of IGBTA. The results of their study showed that simultaneous intervention with IGB and IGBTA had a greater effect in reducing BMI than their separate intervention.^[17]^ Supporting the potential utility of IGBTA injection for obesity treatment, Sanchez Torralvo et al^[18]^ conducted a meta-analysis. However, their findings highlight the limitations of current research, emphasizing the need for well-designed, placebo-controlled studies with larger sample sizes and long-term follow-up. Moreover, Muzaffer et al compared the efficacy of IGB than IGBTA in term of weight loss and they found a significant more weight loss in IGB group than IGBTA group.^[19]^

Intragastric balloon therapy is believed to achieve weight loss through 2 primary mechanisms: restriction and potential modulation of gut motility. Studies suggest that balloons with a volume of 400 mL or more can effectively induce satiety in patients.^[20]^ Furthermore, a delayed gastric emptying time induced by the balloon is proposed as another potential mechanism contributing to weight loss. Studies have demonstrated a correlation between slower gastric emptying and successful weight management.^[20,21]^ Changes in motility and gastric emptying following balloon placement are similar to those seen in refractory gastroparesis. The results of some studies have shown that botulinum toxin injection around the pylorus has been able to improve the gastric emptying rate in gastroparesis patients. Ezzedine et al in a clinical trial found that 100 units botulinum toxin pyloric injection led to a significant improvement in solid phase gastric emptying.^[22]^ Similar findings were reported in other studies.^[23,24]^ These results actually caused this hypothesis in our mind that considering that severe delay in motility and gastric emptying time is 1 of the reasons for the symptoms of IGB intolerance, therefore, botulinum toxin pyloric injection could increase IGB tolerance in a way similar to patients with refractory gastroparesis.

Additionally, IGBTA may affect the pyloric sphincter’s tone, potentially mitigating the obstructive symptoms observed in some patients with IGB intolerance. Moreover, by modulating gastric motility, IGBTA co-therapy may also help to regulate the degree of gastric distention caused by the IGB. This could alleviate discomfort and cramping sensations sometimes experienced with IGB placement alone.^[25–27]^

IGB placement offers a valuable minimally invasive option for weight management in patients with obesity who have failed conservative measures.^[28]^ However, IGB intolerance due to gastrointestinal side effects remains a significant hurdle, with reported rates ranging from 3% to 6%.^[29]^ Premature balloon removal due to intolerance can significantly compromise weight loss goals and limit the overall effectiveness of IGB therapy.^[30,31]^

Our study demonstrates that IGBTA co-therapy has the potential to improve patient tolerance and potentially extend the duration of IGB therapy. This could lead to more significant and sustained weight loss outcomes for a broader range of patients with obesity. Furthermore, for patients who struggle with dietary interventions or are not candidates for surgical procedures, IGBTA co-therapy could offer a valuable bridge therapy, promoting weight loss and potentially improving metabolic health parameters.

This case series provides preliminary evidence supporting the efficacy of BTX-A co-therapy for managing IGB intolerance. However, several limitations must be acknowledged. The retrospective design of the study restricts the ability to establish definitive cause-and-effect relationships between the intervention and observed outcomes. Additionally, the small sample size of 14 patients limits the statistical power and generalizability of the findings, as the results may not represent the broader population. The absence of a control group further complicates the interpretation of results, making it difficult to differentiate the effects of BTX-A from other variables, such as natural symptom resolution or variations in individual patient management. Moreover, the follow-up duration of 6 months may not be sufficient to fully evaluate the long-term efficacy and potential delayed side effects of the therapy. Lastly, as a single-center study conducted within a specialized gastroenterology clinic, the findings may lack external validity and applicability to other clinical settings or patient populations.

Future research should aim to address these limitations by employing randomized controlled trials with larger and more diverse sample sizes, as well as extended follow-up periods. Investigating the effects of BTX-A in various clinical settings and across a broader range of patient demographics will further clarify its potential as a standardized intervention for IGB intolerance.

## 5. Conclusion

This case series provides preliminary evidence supporting the potential of IGBTA co-therapy as a novel and promising intervention to manage IGB intolerance and improve patient tolerance to IGB therapy. Further well-designed studies are needed to confirm these findings and establish IGBTA as a safe and cost-effective strategy for weight management in patients with obesity. If future research confirms the efficacy and safety of IGBTA co-therapy, this approach could significantly expand the applicability of IGB therapy and contribute to the ongoing effort to combat the global obesity epidemic.

Future research is warranted to explore the potential of IGBTA co-therapy on a larger scale. Randomized controlled trials with control groups and standardized outcome measures are necessary to definitively confirm the efficacy and safety of this approach. These studies should also consider including patients with different etiologies of IGB intolerance (pressure vs obstructive effects) to assess the broader applicability of IGBTA co-therapy. Investigating the optimal dosage, injection technique, and long-term durability of IGBTA effects would be valuable for refining this therapeutic strategy and establishing standardized protocols.

## Acknowledgments

We gratefully thank from all of the patients that participated in this study.

## Author contributions

**Conceptualization:** Farzad Faramarzi Garousi, Maryam Ghodsi.

**Data curation:** Mohsen Sedighiyan.

**Investigation:** Farzad Faramarzi Garousi.

**Methodology:** Farzad Faramarzi Garousi, Mohsen Sedighiyan, Maryam Ghodsi.

**Writing – original draft:** Farzad Faramarzi Garousi, Mohsen Sedighiyan.

**Writing – review & editing:** Farzad Faramarzi Garousi.
